# Differentiation of neurogenic tumours and pleomorphic adenomas in the parapharyngeal space based on texture analysis of T2WI

**DOI:** 10.1186/s12903-023-03283-6

**Published:** 2023-08-09

**Authors:** Xuewei Zheng, Chencui Huang, Baoting Yu, Shuo Liu, Tong Li, Yuyao Guan, Jun Ding

**Affiliations:** 1 Department of Radiology, China-Japan Union Hospital of Jilin University, No. 829 of Xinmin Street, Chaoyang District, Changchun, 130021 China; 2 Department of Research Collaboration, R&D Center, Beijing Deepwise and League of PHD Technology Co. Ltd., Beijing, 100080 China

**Keywords:** Parapharyngeal space, Pleomorphic adenoma, Neurogenic tumour, MRI, Texture analysis

## Abstract

**Background:**

The purpose of this study was to identify neurogenic tumours and pleomorphic adenomas of the parapharyngeal space based on the texture characteristics of MRI-T2WI.

**Methods:**

MR findings and pathological reports of 25 patients with benign tumours in the parapharyngeal space were reviewed retrospectively (13 cases with pleomorphic adenomas and 12 cases with neurogenic tumours). Using PyRadiomics, the texture of the region of interest in T2WI sketched by radiologists was analysed. By using independent sample t-tests and Mann‒Whitney U tests, the selected texture features of 36 Gray Level Co-Occurrence Matrix (GLCM) and Gray Level Dependence Matrix (GLDM) were tested. A set of parameters of texture features showed statistically significant differences between the two groups, which were selected, and the diagnostic efficiency was evaluated via the operating characteristic curve of the subjects.

**Results:**

The differences in the three parameters – small dependence low level emphasis (SDLGLE), low level emphasis (LGLE) and difference variance (DV) of characteristics – between the two groups were statistically significant (*P* < 0.05). No significant difference was found in the other indices. ROC curves were drawn for the three parameters, with AUCs of 0.833, 0.795, and 0.744, respectively.

**Conclusions:**

There is a difference in the texture characteristic parameters based on magnetic resonance T2WI images between neurogenic tumours and pleomorphic adenomas in the parapharyngeal space. For the differential diagnosis of these two kinds of tumours, texture analysis of significant importance is an objective and quantitative analytical tool.

There is a potential tissue space known as the parapharyngeal space (PPS) that occupies the upper part of the neck, extending from the base of the skull to the hyoid bone. It is composed of a complex structure. PPS tumours are uncommon and account for less than 1% of all head and neck neoplasms [1]. PPS is anatomically surrounded by several important structures, including the anterior styloid space and posterior styloid space formed by the styloid process. A majority of PPS tumours are benign [2], and pleomorphic adenomas originating from small salivary glands are the most common ones, usually occurring in the anterior styloid septum, followed by neurogenic tumours, usually originating in the posterior styloid septum [3–6]. Only 20% of tumours are malignant [7]. As the tumour is hidden in the parapharyngeal space, the early symptoms may not be apparent. A parapharyngeal space tumour is generally large in size when the patient visits a physician. It is common for the diagnosis to be incorrect due to the difficulty of determining the anatomical location alone. At present, MRI imaging is an effective method for evaluating head and neck diseases. A texture analysis of MRI is a relatively newly developed technology that describes the pixel value features that are invisible to the naked eye, which can illustrate small changes in pixel value. In MRI, texture analysis is a method for the noninvasive differentiation of PPS tumours, a useful technique for the selection of treatment plans and the assessment of prognosis. PPS tumours are treated with surgery as the first option. A variety of surgical approaches have been described in the literature, including the transcervical approach, the parotid approach, the oral approach, and the mandibular approach [8]. In most cases of benign tumours in the parapharyngeal space, the transcervical approach is the most commonly used surgical approach [8–11]. Due to many surgical complications associated with neurogenic tumours, such as nerve injury and massive haemorrhage, surgical risks should be fully explained to patients before surgery.

## Methods

### Patients

From January 2018 to January 2022, 25 cases of PPS tumours (10 males and 15 females) were retrospectively collected, ranging in age from 20 to 79 years old, including **12** cases of neurogenic tumours (8 cases of neurilemmoma, **2** cases of neurofibroma, and **2** cases of paraganglioma), **13** cases of pleomorphic adenoma, 13 cases with lesions occurring on the left side, 12 cases on the right side, 6 patients with anti-inflammatory treatment experiences, and the remaining patients were initial cases. The chief clinical symptoms of all patients included 5 cases of sensation of a foreign body in the pharynx, 5 cases of lateral wall bulge of the oropharynx noticed by patients, 6 cases of painless mass in the upper neck, and 9 cases of pain and dysphagia. Before surgery, MR imaging was performed for every patient. Criteria for inclusion included patients with PPS neurogenic tumour and pleomorphic adenoma confirmed by pathology after surgery, while patients served complete and clear preoperative MR imaging. The criteria for exclusion were as follows: cases in which the largest diameter of the tumour was less than 5 mm, difficult delineation of lesions, no clear surgical records or pathological information, and poor MRI quality. This study fits the point of view in the 1975 Helsinki Declaration, which was revised in 2013 and was approved by the Ethics Committee of China-Japan Union Hospital of Jilin University. All patients signed the informed consent form.

### The method of MRI examination

MRI was performed using a Siemens MagnetomAvanto l.5 T nuclear magnetic resonance machine in coronal, sagittal, and transverse planes. Fast spin echo T1WI and T2WI sequences and fat inhibition and water inhibition T2WI sequences were the scanning sequences. Diffusion-weighted images were obtained in the coronal plane. On the basis of DWI, an ADC diagram was generated. The TR/TE was 7500 ms/80 ms, the FOV was 300 × 320 cm, the matrix size was 256 × 256, the layer thickness was 3 mm, the number of layers was 30, the layer spacing was 1 mm, and the b value was 800 s/mm^2^. All patients underwent a T1WI fat suppression enhancement scan, and 15 ml of gadolinium diamine was administered intravenously. Changes in the lesions following enhancement were used to evaluate the relationships between the tumour and adjacent tissues and blood vessels.

### ROI segmentation of tumours

**Fig. 1:**
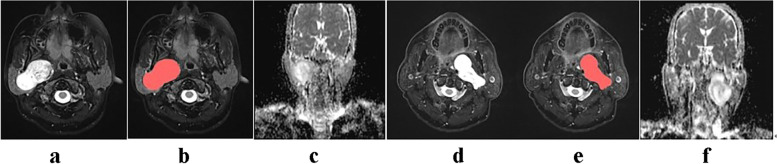
Images** a** and **b** illustrate the sketch of the T2WI axial image of the ROI of a pleomorphic adenoma in the right parapharyngeal space. Image** c** is the ADC diagram corresponding to image** a**. Images **d** and **e** illustrate the sketch of the T2WI axial image of the ROI of neurofibroma in the left parapharyngeal space. Image** f** shows the ADC diagrams that correspond to Image** d**

The high-resolution T2WI original DICOM images of 25 patients were imported into the Shenrui Multimodal Research Platform (http://keyan.deepwise.com, v1.6.2). Two senior imaging physicians drew the ROI layer by layer along the tumour's edge on T2WI images. The ROI showed necrosis, cystic changes, and bleeding components within the tumour (Fig. 1). The computer automatically generated the three-dimensional volume of interest (VOI) of the lesion by avoiding the surrounding oedema. Disagreements between physicians were resolved via discussion.The reproducibility of radiomic features was assessed using intraclass correlation coefficient(ICC). Features with an ICC > 0.75 were selected for statistical analysis.In the neurogenic tumour group and the pleomorphic adenoma group, the ADC values were measured separately, and cystic necrosis and bleeding were avoided.

### Extraction of MRI texture features

A B-spline interpolation resampling was used, and the anisotropic voxels were resampled to form isotropic voxels of 2.0 mm × 2.0 mm. The MRI images were then normalized by centring them at the mean with standard deviation.

$$\mathrm{f }(x)=\frac{s(\mathrm{x }-\mathrm{ \mu x})}{\sigma x}$$

(s = 100; μχ represents the mean value; σ represents the standard deviation).

**Fig. 2:**
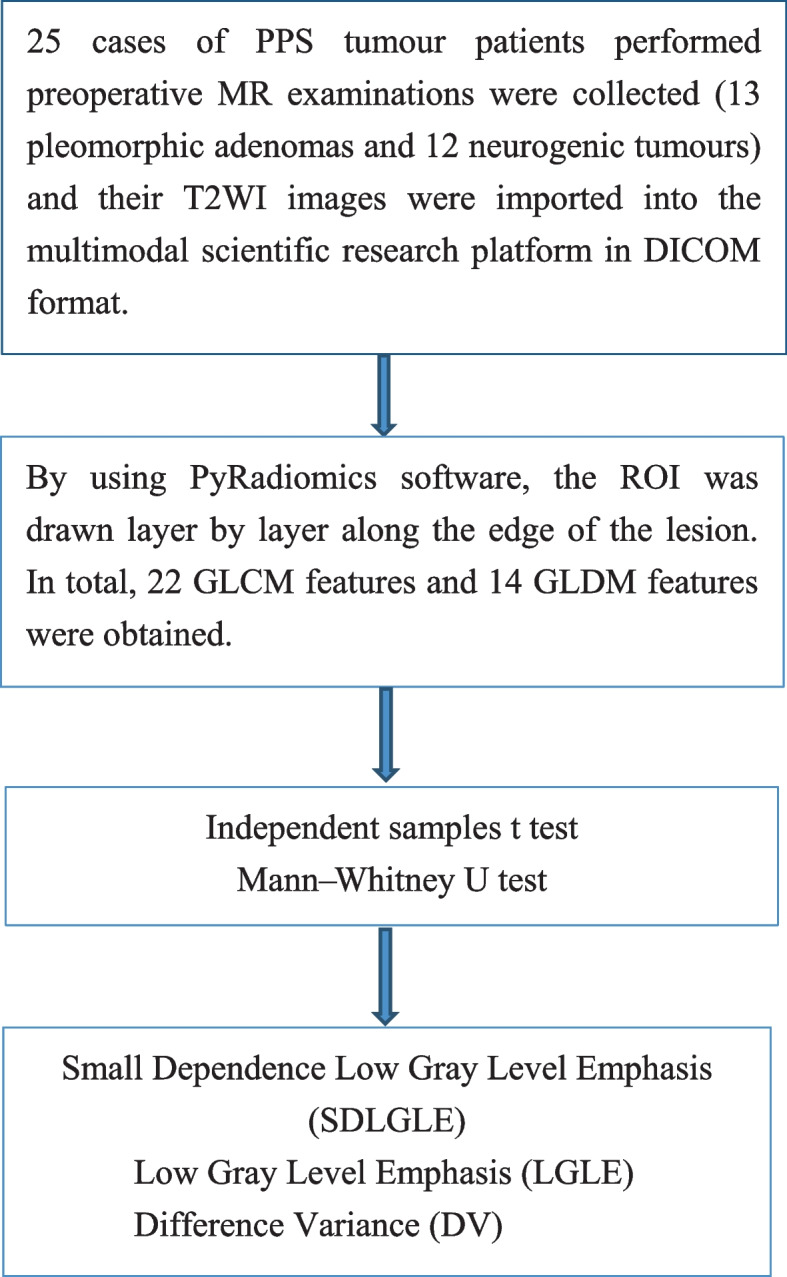
An overview of the process of extracting texture characteristics from MRI images

After obtaining the results of tumour segmentation, two characteristics were extracted from the original T2WI sequence images using the open-source PyRadiomics toolkit software according to the original T2WI sequence images: the 36 texture characteristic parameters comprised 22 characteristics of the GLCM and 14 characteristics of the GLDM (Fig. 2).

### Statistical analysis

The 36 extracted texture feature parameters were statistically analysed using SPSS 22.0 software(IBM Corp.,Armonk, NY, USA). The normal distribution data were expressed as the mean ± standard deviation (‾x ± s) and compared by using the independent samples t test; the nonnormal distribution data were compared using the Mann‒Whitney U test, and the difference was statistically significant (*P* < 0.05). Then, the receiver operating characteristic curve (ROC) was drawn for the statistically significant parameters, and the area under the curve (AUC), sensitivity and specificity were calculated, as well as the effectiveness of the model in predicting neurogenic tumours and pleomorphic adenomas in the parapharyngeal space.

## Results

### Patients' general information

There was no significant difference in age, sex, tumour location, pain level, or maximum tumour diameter among the 25 patients with PPS tumours (as shown in Table 1).

**Table 1:** Clinical and radiological characteristics of patients

Characteristic	All Patients	Neurogenic Tumour	Pleomorphic Adenoma	*P* value
n	25	12	13	
Age(years)	42 ± 15	38 ± 10	45 ± 17	0.205
**Sex**				0.870
Female	15	7	8	
Male	10	5	5	
**Tumour location**				0.255
Left	13	6	7	
Right	12	6	6	
**Pain**				0.053
Yes	9	5	4	
No	16	7	9	
**Maximum diameter(mm)**	44.9 ± 13.4	46.3 ± 13.7	43.7 ± 13.5	0.637
ADC values (*10^**−3**^**mm**^**2**^**/s)**	1.50 ± 0.26	1.51 ± 0.19	1.49 ± 0.32	0.880

### Comparison of texture analysis results

**Fig. 3:**
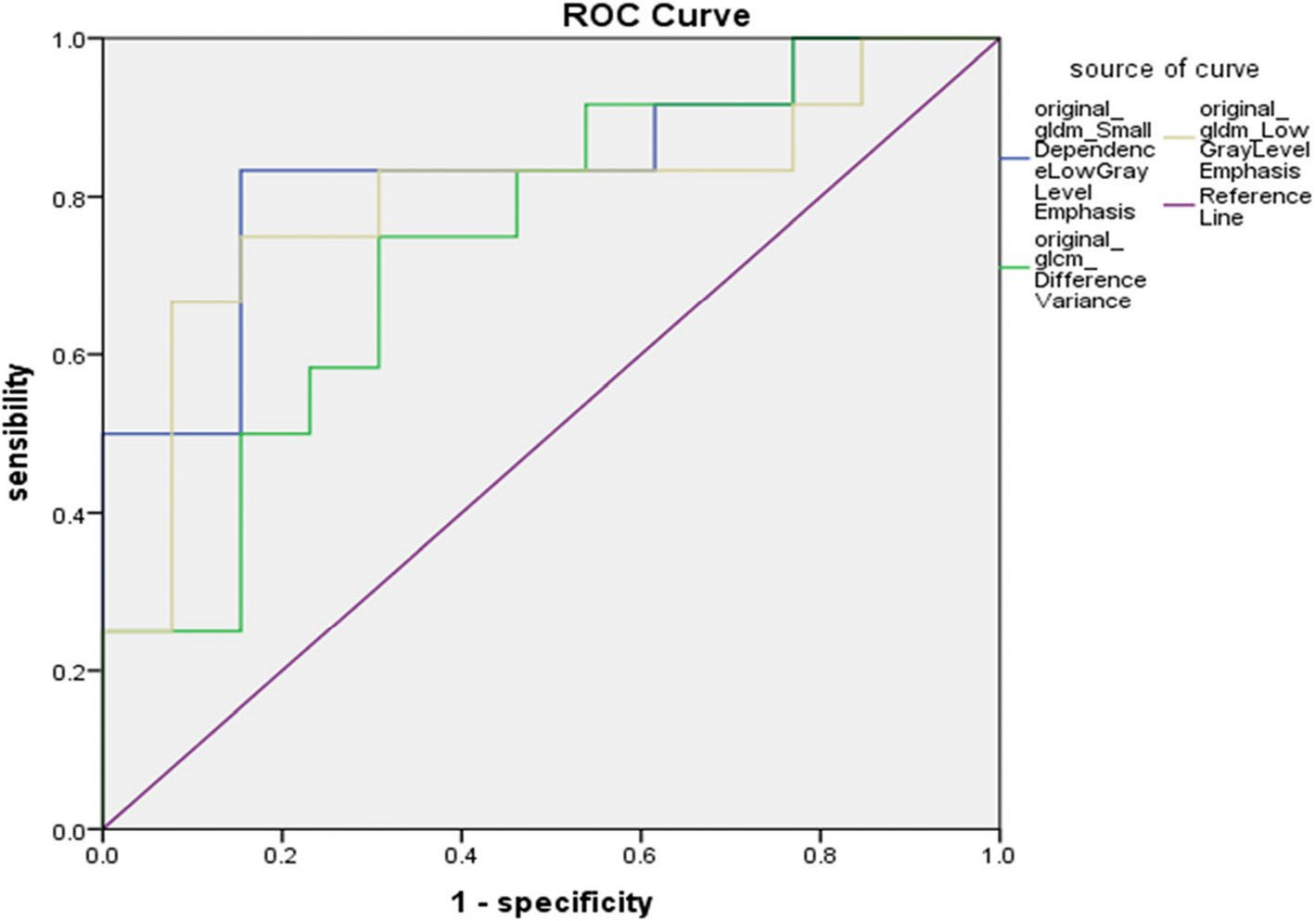
ROC curves of three texture parameters

PyRadiomics software was used to analyse the texture of the original T2WI images of 25 patients.Features showing poor consistency between different groups were removed by calculating the intraclass correlation coefficient,and identify 36 texture characteristics, which contain 22 Gy level co-occurrence matrix features (GLCMs) and 14 Gy level dependent matrix features (GLDMs). In Table 2, three texture characteristics were found to be statistically significant between the PPS neurogenic tumour and pleomorphic adenoma groups (*P* < 0.05). ROC curves were generated for three texture parameters: SDLGLE, LGLE and DV. In Fig. 3, ROC curves for the three parameters are shown, and the area under the curve, the sensitivity, and the specificity were calculated. Based on the three characteristic parameters, SDLGLE had the largest area under the curve, which was 0.833 (Table 3).

**Table 2:** Comparison of the results of three parameters: SDLGLE, LGLE, and DV

Texture Parameter	SDLGLE	LGLE	DV
t/z	-3.288	-2.502^a^	-2.115
P	0.003	0.110	0.045

^a^Represents the nonnormal distribution of data, and the Mann‒Whitney

The U test was used to compare the data between groups

**Table 3:** ROC curve analysis of texture parameters between the neurogenic group tumours and group pleomorphic adenomas in the parapharyngeal space (PPS)

Parameter	AUC	Sensitivity	Specificity	95% confidence interval
SDLGLE	0.833	0.833	0.846	0.666–1.000
LGLE	0.795	0.750	0.846	0.604–0.985
DV	0.744	0.750	0.692	0.549–0.939

## Discussion

The majority of parapharyngeal space tumours are neurogenic tumours, salivary gland tumours and other types. Due to the overlap of certain imaging characteristics, differential diagnosis is complex. A certain misdiagnosis rate is associated with the preoperative diagnosis of tumour origin by routine imaging examination, tumour anatomical location, and tumour relationship to surrounding tissues. According to previous studies, neurogenic tumours are generally located in the posterior space of the styloid process, and the posterior space is large, housing the IX, X, XI, and XII cranial nerves, the sympathetic trunk, internal jugular vein, and internal carotid artery passing through. The tumours are frequently accompanied by cystic areas with clear boundaries, and the arteries and veins move forward or outward. The majority of tumours are neurilemmoma, neurofibroma, and paraganglioma [12, 13]. The other types of PPS tumours include lymphoma, haemangioma, lipoma, teratoma, and metastasis, which account for 20% of PPS tumours [14]. The anterior styloid space is small, including the external carotid artery, ascending pharyngeal artery, deep lobe of the parotid gland, and a small salivary gland. Parotid gland tumours of the deep lobe and ectopic salivary gland tumours are therefore frequently found in this space. Approximately 80%-90% of salivary gland tumours are pleomorphic adenomas, and 10–20% of them are malignant mucoepidermoid carcinomas [15]. Because the anatomical structure of PPS is complicated and the incidence of tumours is relatively low, it is crucial to select the optimal surgical method by determining the precise location and origin of the PPS tumour, such as the transcervical approach, the parotid approach, the oral approach, and the combined approach. Historically, PPS tumours were diagnosed by dividing the PPS into two parts (the anterior and posterior styloid processes) and using the location of the internal carotid artery (ICA) as a diagnostic marker [16]. Through the forward or backwards displacement of the ICA, it was determined whether the tumours in the anterior styloid process space originated from parotid gland tumours and ectopic salivary gland tumours or whether the tumours in the posterior styloid process space were predominantly neurogenic. Liu [17] et al. suggested that judging PPS tumours based solely on ICA displacement has limitations. Since the ICA has the structure of the posterior styloid space, tumours from tissues outside the carotid sheath of the posterior styloid space and nasopharyngeal tumours invading the styloid process may cause the ICA to move backwards, whereas some tumours may surround the ICA without causing it to move. A retrospective analysis revealed that 8 of the 12 neurogenic tumours in this study caused arteriovenous displacement. Ten cases of neurogenic tumours were found in the posterior space of the styloid process. Only 1 of 13 pleomorphic adenomas caused arteriovenous displacement, and all thirteen tumours were found in the anterior space of the styloid process. Therefore, judging PPS tumours solely by their positions is unreliable. This study's findings are generally consistent with Liu's [17]. In the previous domestic and international literature, the objective and quantitative identification of PPS tumours by combining MR texture characteristics has not been discussed.

Texture analysis is a novel method of analysing medical images. It is the extraction of texture-specific parameters using specific image processing techniques to obtain quantitative and qualitative descriptions of texture [18, 19]. Noninvasive methods are used to evaluate the internal characteristics of tumours for diagnosis, treatment, and prognosis [20]. Texture analysis has emerged in numerous medical fields in recent years, including the differential diagnosis of benign and malignant mucinous soft tissue tumours [21], the prediction of the pathological grade of breast phyllodes tumours [22], and research on the differentiation and grade of cervical squamous cell carcinoma [23]. Basic first-order statistics are used to describe the pixel intensity and its distribution in the target area as the first-order characteristics of texture. Local texture features of two adjacent pixels are calculated by second-order characteristics. Gray Level Co-Occurrence Matrix (GLCM), Gray Level Run Length Matrix (GLRLM), Gray Level Size Zone Matrix (GLSZM), Gray Level Dependence Matrix (GLDM), Neighborhood Gray Tone Difference Matrix (NGTDM**)**, etc., are examples of common methods. High-order image characteristics analyse localized image data [24]. Drabycz et al. [25] believed that T2WI in MRI can extract texture characteristics more effectively. However, in this study, the texture characteristic parameters were extracted from T2WI. It was discovered that SDLGLE, LGLE, and DV are statistically significant (*P* < 0.05) in distinguishing neurogenic tumours and pleomorphic adenomas in the parapharyngeal space, among which SDLGLE measures the joint distribution of small dependence with lower gray-level values. LGLE measures the distribution of low gray-level values, with a higher value indicating a greater concentration of low gray-level values in the image. Difference variance is a measure of heterogeneity that places higher weights on differing intensity level pairs that deviate more from the mean. The mean values and standard deviations of the three parameters SDLGLE, DV, and LGLE were higher in the neurogenic tumour group than in the pleomorphic adenoma group (*P* < 0.05). SDLGLE has the highest diagnostic efficiency, with an AUC of 0.833, a sensitivity of 83.3% and a specificity of 84.6%. In this study, the respective ADC values of the two groups of tumours were determined. The ADC values for the neurogenic tumour group were found to be (1.51 ± 0.19) * 10^–3^ mm^2^/s, while those for the pleomorphic adenoma group were (1.49 ± 0.32) * 10^–3^ mm^2^/s. There was no statistically significant difference between the ADC values of the two groups. Therefore, the degree of tumour cell diffusion limitation cannot be used as a diagnostic index. In this paper, the advantages of the quantitative description of tumours by MRI texture analysis are gradually emerging, and the origin of tumours in the parapharyngeal space is determined by a noninvasive preoperative examination method to guide clinics in the correct diagnosis and treatment.

### Limitations

First, parapharyngeal space tumours are extremely rare. Due to the small sample size of our research, our findings may not be generalizable. Second, in the future, we will be able to study multiple sequence textures, including T2WI and DWI joint sequences.

## Conclusions

Through texture analysis of MRI T2WI, we discovered that neurogenic tumours and pleomorphic adenomas in the parapharyngeal space differ in three texture parameters: SDLGLE, LGLE, and DV. These parameters are found in the gray level co-occurrence matrix and gray level dependence matrix. They compensate for the deficiency of the previous diagnosis based solely on the anatomical location of the tumours and perform a quantitative evaluation of the two tumours, thereby guiding the clinically scientific and individually tailored treatment.

## Data Availability

All data generated or analysed during this study are included in this published article.

## Abbreviations

MRI: Magnetic resonance imaging
GLCM: Gray Level Co-Occurrence Matrix
GLDM: Gray Level Dependence Matrix
SDLGLE: Small Dependence Low Gray Level Emphasis
LGLE: Low Gray Level Emphasis
DV: Difference Variance
DWI: Diffusion-weighted imaging
ADC: Apparent diffusion coefficient
VOI: Volume of interests
ROI: Region of interest
PPS: Parapharyngeal space
ICA: Internal carotid artery
GLRLM: Gray Level Run Length Matrix
GLSZM: Gray Level Size Zone Matrix
NGTDM: Neighborhood Gray Tone Difference Matrix

## References

[1] Marzouki H, Nujoom M, Fagih SN, Almokri RM, Zawawi F, Jamjoom R, Almarzouki HZ, Merdad M (2022). Surgical parapharyngeal space tumor analysis with case series study. Comput Intell Neurosci.

[2] Pang KP, Goh CH, Tan HM (2002). Parapharyngeal space tumours: an 18 year review. J Laryngol Otol.

[3] Kuet ML, Kasbekar AV, Masterson L, Jani P (2015). Management of tumors arising from the parapharyngeal space: a systematic review of 1,293 cases reported over 25 years. Laryngoscope.

[4] Saito DM, Glastonbury CM, El-Sayed IH (2007). Parapharyngeal space schwannomas: preoperative imaging determination of the nerve of origin. Arch Otolaryngol Head Neck Surg.

[5] Anil G, Tan TY (2011). CT and MRI evaluation of nerve sheath tumors of the cervical vagus nerve. AJR Am J Roentgenol.

[6] Shirakura S, Tsunoda A, Akita K (2010). Parapharyngeal space tumors: anatomical and image analysis findings. Auris Nasus Larynx.

[7] Vallabhaneni AC, Mandakulutur SG, Vallabhaneni S, Prabha A, Banavara RK. True parapharyngeal space tumors: case series from a teaching Oncology center. Indian J Otolaryngol Head Neck Surg. 2017;69(2):225–9. 10.1007/s12070-017-1099-0 PMC5446346 28607895

[8] Bradley PJ, Bradley PT, Olsen KD (2011). Update on the management of parapharyngeal tumours. Curr Opin Otolaryngol Head Neck Surg.

[9] Jbali S, Khaldi A, Touati S, Gritli S (2021). Surgical approaches to parapharyngeal space tumors: an example and review of the Literature. Case Rep Surg.

[10] Malone JP, Agrawal A, Schuller DE (2001). xcSafety and efficacy of transcervical resection of parapharyngeal space neoplasms. Ann Otol Rhinol Laryngol.

[11] EiseleDW, Richmon JD. Contemporary evaluation and management of parapharyngeal space neoplasms. J Laryngol Otol, 2013;127(6): 550–555. 10.1017/S0022215113000686 23575439

[12] Joshi P, Joshi KD, Nair S, Bhati M, Nair D, Bal M, Joshi A, Mummudi N, Tuljapurkar V, Chaukar DA, Chaturvedi P (2021). Surgical management of parapharyngeal tumors: our experience. South Asian J Cancer.

[13] Matsuki T, Miura K, Tada Y, Masubuchi T, Fushimi C, Kanno C, Takahashi H, Kamata S, Okamoto I, Miyamoto S, Yamashita T (2019). Classification of tumors by imaging diagnosis and preoperative fine-needle aspiration cytology in 120 patients with tumors in the parapharyngeal space. Head Neck.

[14] Bozza F, Vigili MG, Ruscito P, Marzetti A, Marzetti F. Surgical management of parapharyngeal space tumours:results of 10-year follow-up. Acta Otorhinolaryngol Ital. 2009;29(1):10–15. PMC2689563 19609376

[15] Tincani AJ, Martins AS, Altemani A (1999). Parapharyngeal space tumors: considerations in 26 cases. Sao Paulo Med J.

[16] Som PM, Biller HF, Lawson W (1984). Parapharyngeal space masses: an updated protocol based upon 104 cases. Radiology.

[17] Liu XW, Wang L, Li H, Zhang R, Geng ZJ, Wang DL, Xie CMA. modified method for locating parapharyngeal space neoplasms on magnetic resonance images: implications for differential diagnosis. Chin J Cancer. 2014;33(10):511–520. 10.5732/cjc.014.10017 PMC4198754 25104280

[18] Al-Kadi OS, Watson D (2008). Texture analysis of aggressive and nonaggressive lung tumor CE CT images. IEEE Trans Biomed Eng.

[19] Speckter H, Bido J, Hernandez G (2018). Pretreatment texture analysis of routine MR images and shape analysis of the diffusion tensor for prediction of volumetric response after radiosurgery for meningioma. J Neurosurg.

[20] Gillies RJ, Kinahan PE, Hricak H (2016). Radiomics: images are more than pictures, they are data. Radiology.

[21] Chang H, Kang Y, Ahn JM, Lee E, Lee JW, Kang HS (2022). Texture analysis of magnetic resonance image to differentiate benign from malignant myxoid soft tissue tumors: a retrospective comparative study. PLoS One.

[22] Mao Y, Xiong Z, Wu S, Huang Z, Zhang R, He Y, Peng Y, Ye Y, Dong T, Mai H (2022). The predictive value of magnetic resonance imaging-based texture analysis in evaluating histopathological grades of breast phyllodes tumor. J Breast Cancer.

[23] Shi B, Dong JN, Zhang LX, Li CP, Gao F, Li NY, Wang CB, Fang X, Wang PP. A combination analysis of IVIM-DWI Biomarkers and T2WI-based texture features for tumor differentiation grade of cervical squamous cell carcinoma. Contrast Media Mol Imaging. 2022; 2022:2837905. 10.1155/2022/2837905 PMC8947887 35360261

[24] Chicklore S, Goh V, Siddique M (2013). Quantifying tumour heterogeneity in 18F-FDG PET/CT imaging by texture analysis. Eur J Nucl Med Mol Imaging.

[25] Drabycz S, RoldánG, de Robles P, et al. An analysis of image texture, tumor location, and MGMT promoter methylation in glioblastoma using magnetic resonance imaging. Neuroimage. 2010;49(2):1398–1405. 10.1016/j.neuroimage.2009.09.049 19796694

